# Psychiatric and medical admissions observed among elderly patients with new-onset epilepsy

**DOI:** 10.1186/1472-6963-11-84

**Published:** 2011-04-19

**Authors:** Laurel A Copeland, Alan B Ettinger, John E Zeber, Jodi M Gonzalez, Mary Jo Pugh

**Affiliations:** 1 Veterans Affairs: Central Texas Veterans Health Care System (CAHR), 2102 Birdcreek Dr, Temple, TX 76502 USA; 2 Scott & White Healthcare, Center for Applied Health Research, Temple, TX USA; 3 Neurological Surgery, P.C., Lake Success, NY USA; 4 University of Texas Health Science Center at San Antonio, Department of Psychiatry, San Antonio, TX USA; 5 Veterans Affairs HSR&D: South Texas Veterans Health Care System (VERDICT), 7400 Merton Minter (11c6), San Antonio, TX 78229-4404 USA; 6 University of Texas Health Science Center at San Antonio, Department of Epidemiology & Biostatistics, San Antonio, TX USA

## Abstract

**Background:**

Inpatient utilization associated with incidence of geriatric new-onset epilepsy has not been characterized in any large study, despite recognized high levels of risk factors (comorbidity).

**Methods:**

Retrospective study using administrative data (Oct '01-Sep '05) from the Veterans Health Administration from a nationwide sample of 824,483 patients over age 66 in the retrospective observational Treatment In Geriatric Epilepsy Research (TIGER) study. Psychiatric and medical hospital admissions were analyzed as a function of patient demographics, comorbid psychiatric, neurological, and other medical conditions, and new-onset epilepsy.

**Results:**

Elderly patients experienced a 15% hospitalization rate in FY00 overall, but the subset of new-onset epilepsy patients (n = 1,610) had a 52% hospitalization rate. New-onset epilepsy was associated with three-fold increased relative odds of psychiatric admission and nearly five-fold increased relative odds of medical admission. Among new-onset epilepsy patients, alcohol dependence was most strongly associated with psychiatric admission during the first year after epilepsy onset (odds ratio = 5.2; 95% confidence interval 2.6-10.0), while for medical admissions the strongest factor was myocardial infarction (odds ratio = 4.7; 95% confidence interval 2.7-8.3).

**Conclusion:**

From the patient point of view, new-onset epilepsy was associated with an increased risk of medical admission as well as of psychiatric admission. From an analytic perspective, omitting epilepsy and other neurological conditions may lead to overestimation of the risk of admission attributable solely to psychiatric conditions. Finally, from a health systems perspective, the emerging picture of the epilepsy patient with considerable comorbidity and demand for healthcare resources may merit development of practice guidelines to improve coordinated delivery of care.

## Background

Epilepsy is a common neurologic disorder among persons over age 65, with a prevalence estimated at 5.7 cases per 1,000 older adults [1,2]. Incidence may range from 16 to 51 cases per 100,000 in population-based studies [3]. While the diagnosis and treatment of seizures in this population is a challenge in its own right, comorbid conditions may lead to hospital admissions for reasons that go beyond epilepsy. Comorbidities with elevated occurrence among epilepsy patients include digestive system disorders, stroke, respiratory disorders, dementia, migraine and other pain conditions as well as psychiatric disorders [3-5]. If the risk and nature of non-epilepsy hospitalizations are substantial, this means that elderly new-onset epilepsy patients are a special population in whom clinicians should pay careful attention to identifying and addressing comorbidity[6]. In this context, epilepsy becomes a marker for a constellation of serious conditions that go substantially beyond the epilepsy itself. Yet little is known about the rate and nature of hospitalizations among elderly patients with new-onset epilepsy.

Previous research on older epilepsy patients treated in the VA noted a large burden of psychiatric disorders,[7] which may contribute directly to inpatient stays. Seizure disorders, other medical conditions including cerebrovascular disorders,[5] or psychiatric conditions may necessitate inpatient admission among elderly patients with epilepsy [8]. These inpatient experiences have rarely been described outside economic analyses and studies of emergency department use [9-11].

As part of the Treatment In Geriatric Epilepsy Research (TIGER) study of new-onset late-life epilepsy, we examined risk factors for psychiatric and medical admissions among geriatric patients, comparing those with new-onset epilepsy to those without epilepsy, expecting that TIGER patients would have higher relative odds of admission. Next we analyzed admissions of new-onset epilepsy patients only, to better understand risk factors for medical and psychiatric hospitalization in that clinically unique subset of patients.

## Methods

### Study design

The TIGER study is a national retrospective, observational study of new-onset epilepsy in geriatric patients receiving care in the Veterans Health Administration (VA).

### Sample

The sample consisted of VA patients aged 66 or older in fiscal year 2000 (FY2000; Oct 1999-Sep 2000). The prior two years were used to establish epilepsy status as no epilepsy, ongoing epilepsy, or new-onset epilepsy. Based on a validated algorithm comparing chart review data to administrative and Medicare ICD-9 codes and electronic medication records, both diagnosis of a seizure disorder (ICD-9 code 780.3 or 345) and a current anti-epileptic drug (AED's) prescription were required to establish epilepsy status. The algorithm has a positive predictive value of 0.98 [12]. New-onset epilepsy was defined by use of the VA without diagnosis of a seizure disorder in FY1998-FY1999, and diagnosis of seizure disorder plus receipt of AED's in FY2000 but not in FY1998-FY1999. Patients with previous diagnosis of epilepsy (prevalent cases) were excluded (n = 41,867). This study was approved by the Institutional Review Boards at the University of Texas Health Science Center at San Antonio and at the Bedford and Hines VA Medical Centers prior to initiation.

### Data sources

Administrative data extracts of the VA's all-electronic medical record provided measures of diagnoses, inpatient and outpatient utilization, and demographics.

### Measures

The years FY1998-FY1999 were also used to establish neurological conditions associated with seizure disorders present prior to determining epilepsy onset. These "prior" conditions included diagnosis of senile dementia per Krishnan,[13] cerebrovascular disorders (ICD-9 codes 430-438), brain tumor (ICD-9 191), head injury (ICD-9 851-854), and other neurological conditions such as multiple sclerosis and Parkinsons [14].

Then administrative data extracts determined hospitalizations during the first year (FY2000) and comorbid conditions, while the second year (FY2001) established ongoing use of the VA healthcare system (yes or no). Sociodemographic measures included age group (young-old 65-74 years of age, older 75-84, oldest 85+), ethnicity/race (African-American or Hispanic versus white), and gender. Patients missing data on race were identified and excluded (20% of sample).

The predictors of interest were demographic measures, prior neurological conditions, other comorbid medical conditions, psychiatric conditions, and new-onset epilepsy. Selim comorbidity indicators, developed on VA patient survey data and subsequently adapted to administrative data[15,16] were employed to identify the comorbid medical and psychiatric conditions. The Selim indicators assess diagnoses for diabetes, hypertension, myocardial infarct, angina, arrhythmia, congestive heart failure, and transient ischemic attacks, among other conditions. The Selim indicator of seizures was not included in analytic models as these diagnosis codes were incorporated into the new-onset epilepsy indicator, leaving 29 Selim physical comorbidity indicators in the model. Psychiatric indicators assessed diagnosis with schizophrenia (ICD-9 295), bipolar disorder (ICD-9 296.0-296.1, 296.4-296.8), depressive disorder (ICD-9 296.2-296.3, 300.4, 311), post-traumatic stress disorder (ICD-9 309.81), alcohol dependence (ICD-9 303, 305.0), and anxiety disorders (ICD-9 300.00, 300.02, 300.09),[15] to which was added an indicator of substance use disorder (ICD-9 291, 292, 303-305).

Outcomes were hospital admission events (any versus none). Psychiatric admissions were identified by a hospitalization with a primary diagnosis of a psychiatric disorder in the range reviewed above including those not specified (ICD-9 codes 290-311). Psychiatric diagnoses in the range ICD-9 312-319 are related to childhood/mental retardation and are extremely rare in the post-military population that uses the VA. There were 25 persons with admissions attributed to this range of diagnosis codes; these individuals were excluded from the study, leaving a sample size of 824,483 persons. Medical admissions were all those with a primary diagnosis outside the range 290-319. Epilepsy-specific admissions, a subset of medical admissions, were admissions with a primary diagnosis of a seizure disorder (ICD-9 345, 780.39). We report these separately to establish that medical admissions of the new-onset epilepsy patients were not solely related to their epilepsy.

### Analysis

After characterizing the sample with descriptive statistics, adjusted logistic regression models assessed factors in admission (yes/no) in FY2000, including demographic measures, the indicators of prior neurological conditions, clinical covariates (medical and psychiatric comorbidities) to adjust for between-group differences, and new-onset epilepsy. Separate models assessed each outcome: psychiatric admission and medical admission. A second set of models restricted to the subset of patients with new-onset epilepsy determined factors in hospitalization specific to TIGER patients. All models included an indicator of loss to follow-up, which identified patients for whom no data was found in FY2001. This included patients who died in FY2001, left the VA system, or remained hospitalized past the end of the observation period at the end of FY2001. In analysis, many modest effects achieve statistical significance when the sample is very large as in this study, therefore we focused on odds ratios indicating medium (≥1.5 for positive associations and ≤0.67 for inverse associations) or large (≥2 or of ≤.5) effect sizes [17]. Model fit was reported by the c-statistic, a measure which ranges from 0.5 for model fit no better than by chance to 1.0 for perfect fit.

## Results

### Sample

The TIGER study of 824,483 patients aged 66 or older in FY2000 included 16,814 women (2%). Consistent with U. S. military recruitment trends, most patients were Anglo males with 12% African-American and 5% Hispanic patients (Table 1). There were 1,610 patients with new-onset epilepsy (TIGER patients) in the sample (0.2%). TIGER patients resembled non-epilepsy patients on most demographic measures (age, gender) but differed in that they were more likely to be African-American (20% of TIGER patients versus 12% of non-TIGER patients, chi-square = 115.6, df = 2, p < .0001). Summing the included and excluded patients for the denominator and adjusting for the deleted cases missing data on race, the incidence of epilepsy in late-life VA patients was 156 cases per 100,000 patients while prevalence was 42 per 1,000.

**Table 1:** Description of Elderly Patients in the Veterans Health Administration in FY2000

Characteristic	TIGER Cohort with New-Onset Epilepsy	Non-Epilepsy Patients	Total Sample
Number of patients	1,610	822,873	824,483

	N (%) or Mean (SD)	N (%) or Mean (SD)	N (%) or Mean (SD)
Age			
65-74 Years	850 (53%)	448,274 (54%)	449,124 (54%)
75-84 Years	706 (44%)	345,985 (42%)	346,691(42%)
85 or older	54 (3%)	28,614 (3%)	28,668 (3%)
Ethnicity/Race			
Hispanic	83 (5%)	44,612 (5%)	44,695 (5%)
African-American	323 (20%)	94,669 (12%)	94,992 (12%)
Anglo	1,204 (75%)	683,592 (83%)	684,796 (83%)
Gender			
Women	24 (1.5%)	16,790 (2%)	16,814 (2%)
Men	1,586 (98.5%)	806,083 (98%)	807,669 (98%)
			
Neurological conditions, other	150 (9%)	32,114 (4%)	32,264 (4%)
Cerebrovascular disease	639 (40%)	136,306 (17%)	136,945 (17%)
Dementia	284 (18%)	60,588 (7%)	60,872 (7%)
Brain tumor	27 (2%)	5,306 (1%)	5,333 (1%)
Head injury	25 (2%)	3,565 (<1%)	3,590 (<1%)
			
Any psychiatric condition	801 (50%)	199,480 (24%)	200,281 (24%)
Anxiety	152 (9%)	43,292 (5%)	43,444 (5%)
Depression	280 (17%)	71,623 (9%)	71,903 (9%)
Post-traumatic stress disorder	77 (5%)	22,382 (3%)	22,459 (3%)
Bipolar disorder	47 (3%)	9,729 (1%)	9,776 (1%)
Schizophrenia	80 (5%)	15,954 (2%)	16,034 (2%)
Alcohol dependence	104 (6%)	16,922 (2%)	17,026 (2%)
Substance abuse	211 (13%)	55,554 (7%)	55,765 (7%)
			
Hypertension	1,147 (71%)	510,382 (62%)	511,529 (62%)
Dyslipidemia	449 (28%)	265,785 (32%)	266,234 (32%)
Diabetes	497 (31%)	221,810 (27%)	222,307 (27%)
			
FY00 psychiatric admission	91 (6%)	7,739 (1%)	7,830 (1%)
FY00 medical admission	809 (50%)	115,548 (14%)	116,357 (14%)
FY00 epilepsy-related admission (a subset of medical admissions)	263 (16%)	4 (<1%)	267 (<1%)

### Comorbidity

New-onset epilepsy patients had high rates of the prior neurological conditions studied: cerebrovascular disease (40% TIGER versus 17% other VA patients over age 66), dementia (18% versus 7%), brain tumor (2% versus 1%) and head injury (2% versus <1%). Psychiatric comorbid conditions were also common, especially depressive disorders which were diagnosed in 17% of new-onset epilepsy patients compared to 9% of non-epilepsy patients. Overall, these older patients averaged 3.0 (± 2.0) Selim physical comorbid conditions of 29 assessed conditions and 0.22 (± 0.56) Selim mental illnesses of six assessed.

### Outcomes

The proportion of geriatric patients with a hospital admission varied by new-onset epilepsy status. Overall, the proportion hospitalized was 15% (121,118) in FY00 but it was 52% (844) for the subset of new-onset epilepsy patients. Most patients had medical admissions (14%; n = 116,357) while 1% (7,830) experienced psychiatric admissions; for TIGER patients these proportions were 50% (809) and 6% (91). Some patients had both types of admissions: 0.4% (3,069) of the large sample and 3.5% (56) of the new-onset epilepsy patients. Among TIGER patients, roughly one-third with medical admissions were admitted specifically for epilepsy as the primary diagnosis for the hospitalization (ICD9 codes 345.xx or 780.39). Only 19 TIGER patients had both psychiatric and epilepsy-specific admissions. The next year in FY01, 14% of all patients (106,483) were admitted as were 29% of new-onset epilepsy patients (387). TIGER patients were more likely to be lost from the study (chi-square 59.4, d.f. = 2, p < .0001), primarily through death.

Days of hospitalization for medical admissions averaged 19.5 days (± 29.8; median 9 days) for the entire sample, and were markedly higher for TIGER patients (median 14.0 vs 9.0 days; Wilcoxon 2-sample z-statistic = 10.4; two-sided p < .0001). Individuals with new-onset epilepsy had somewhat more inpatient days for psychiatric reasons compared to other patients: mean 52.7 (± 58.7) versus 40.8 (± 47.3), and median 32 vs 24 days (Wilcoxon 2-sample z-statistic = 2.03; two-sided p = .04).

In the covariate-adjusted model, psychiatric hospital admission was primarily associated with psychiatric disorders for these geriatric patients but also showed strong associations with prior neurological conditions: cerebrovascular disease (OR = 1.5, 95% CI 1.4-1.6), dementia (OR = 9.5, 95% CI 9.0-10.0), alcohol dependence (OR = 11.7; 95% CI 10.9-12.5), schizophrenia (OR = 6.3; 95% CI 5.9-6.7), bipolar disorder (OR = 4.6; 95% CI 4.2-5.0), depression (OR = 2.8; 95% CI 2.7-3.0). New-onset epilepsy was also associated with increased risk of psychiatric admission (OR = 2.9, 95% CI 2.2-3.7). An inverse relationship with psychiatric admission was noted for Hispanic ethnicity (OR = 0.58, 95% CI 0.51-0.65). Some physical conditions had modest associations, with hepatitis strongest among these (OR = 1.5; 95% CI 1.3-1.7). Complete multivariable results are reported in Table 2.

**Table 2:** Factors Associated with Psychiatric Admissions among Geriatric Patients (N = 824,483; c-statistic = 0.95)

Effect	Odds Ratio	95% CI	Significant (CI excludes 1.0)
Alcohol dependence	11.70	10.9-12.5	*
Dementia	9.50	9.00-10.0	*
Schizophrenia	6.29	5.87-6.73	*
Bipolar disorder	4.59	4.23-4.97	*
New-onset epilepsy	2.87	2.24-3.67	*
Depression	2.83	2.68-2.99	*
Lost to follow-up	2.01	1.89-2.14	*
PTSD	1.67	1.52-1.82	*
Cerebrovascular disease	1.50	1.42-1.59	*
Hepatitis	1.46	1.27-1.68	*
Neurological conditions, other	1.42	1.32-1.53	*
Anemia	1.40	1.32-1.50	*
Anxiety	1.40	1.30-1.50	*
COPD	1.38	1.30-1.45	*
Peptic ulcer	1.38	1.24-1.53	*
Age 85 or older	1.37	1.23-1.52	*
Brain tumor	1.30	1.03-1.65	*
Irregular heartbeat	1.24	1.17-1.33	*
Heart attack	1.23	1.12-1.36	*
Female	1.22	1.05-1.43	*
Head injury	1.22	1.00-1.49	*
African American	1.21	1.13-1.30	*
Hypertension	1.21	1.15-1.28	*
Low-back pain	1.18	1.10-1.27	*
Other arthritic disease	1.17	1.05-1.29	*
Enlarged prostate	1.16	1.10-1.23	*
Hip problems	1.11	0.96-1.27	n.s.
Prostatitis	1.10	0.90-1.35	n.s.
Osteoarthritis	1.07	1.00-1.13	*
Gallbladder disease	0.98	0.79-1.21	n.s.
Gout	0.98	0.87-1.11	n.s.
Peripheral vascular disease	0.97	0.89-1.05	n.s.
Angina	0.95	0.86-1.05	n.s.
Diverticulitis	0.95	0.84-1.08	n.s.
Congestive Heart Failure	0.94	0.87-1.01	n.s.
Age 75-84 years	0.92	0.87-0.97	*
Cancer	0.84	0.79-0.90	*
Rheumatoid arthritis	0.84	0.68-1.03	
Cataracts	0.80	0.75-0.86	*
Inflammatory bowel disease	0.64	0.43-0.96	*
Hispanic	0.58	0.51-0.65	*

In the model of medical admissions among 824,483 geriatric patients, significant factors were noted in each domain. The demographic factor of African American race was associated with greater risk of medical admission (OR = 1.5; 95% CI 1.4-1.5). Among prior neurological conditions, new-onset epilepsy imparted 5-fold increased relative odds (OR = 4.8; 95% CI 4.3-5.5). Other neurologic risk factors roughly doubled relative odds of medical admission: dementia (OR = 2.2; 95% CI 2.1-2.3), head injury (OR = 2.2; 95% CI 2.0-2.4), brain tumor (OR = 1.9; 95% CI 1.8-2.1), cerebrovascular disease (OR = 1.8; 95% CI 1.7-1.8), and other neurological disorders such as multiple sclerosis and Parkinsons (OR = 1.8; 95% CI 1.8-1.9). Many other physical ills were associated with medical admission, notably comorbid gall bladder disorders (OR = 7.4; 95% CI 6.9-7.8), myocardial infarct (OR = 4.7; 95% CI 4.5-4.8), angina (OR = 3.5; 95% CI 3.4-3.6), and anemia (OR = 3.2; 95% CI 3.1-3.3). Among mental disorders, significant associations with medical admission were found for alcohol dependence (OR = 2.3; 95% CI 2.2-2.4) and schizophrenia (OR = 1.5; 95% CI 1.4-1.6; Table 3).

**Table 3:** Factors Associated with Medical Admissions among Geriatric Patients (N = 824,483; c-statistic = 0.86)

Effect	Odds Ratio	95% CI	
Gallbladder disease	7.37	6.94-7.82	*
New-onset epilepsy	4.84	4.29-5.46	*
Heart attack	4.68	4.55-4.81	*
Lost to follow-up	3.74	3.67-3.82	*
Angina	3.51	3.42-3.60	*
Anemia	3.20	3.14-3.26	*
Hip problems	2.75	2.63-2.88	*
Irregular heartbeat	2.59	2.54-2.64	*
Cancer	2.45	2.41-2.49	*
COPD	2.41	2.37-2.45	*
Hepatitis	2.38	2.25-2.51	*
Alcohol dependence	2.30	2.21-2.40	*
Congestive Heart Failure	2.21	2.17-2.25	*
Dementia	2.20	2.15-2.25	*
Head injury	2.19	2.01-2.38	*
Peptic ulcer	1.97	1.91-2.04	*
Brain tumor	1.93	1.80-2.07	*
Inflammatory bowel disease	1.92	1.76-2.10	*
Peripheral vascular disease	1.83	1.79-1.87	*
Neurological conditions, other	1.81	1.75-1.87	*
Cerebrovascular disease	1.75	1.72-1.78	*
Diverticulitis	1.73	1.68-1.79	*
Prostatitis	1.55	1.46-1.64	*
Hypertension	1.48	1.45-1.50	*
Schizophrenia	1.48	1.41-1.55	*
Rheumatoid arthritis	1.47	1.40-1.54	*
African American	1.45	1.42-1.48	*
Enlarged prostate	1.40	1.37-1.42	*
Female	1.39	1.32-1.47	*
Gout	1.33	1.29-1.38	*
Other arthritic disease	1.30	1.26-1.34	*
Low-back pain	1.26	1.23-1.28	*
Depression	1.24	1.21-1.27	*
Hispanic	1.20	1.16-1.24	*
Osteoarthritis	1.14	1.12-1.16	*
Anxiety	1.12	1.09-1.16	*
Age 85 or older	1.11	1.07-1.16	*
Cataracts	1.10	1.08-1.12	*
Bipolar disorder	1.07	1.01-1.14	*
Age 75-84 years	0.90	0.89-0.92	*
PTSD	0.82	0.79-0.86	*

For the subset of 1,610 new-onset epilepsy patients, the model of psychiatric admissions found an effect of prior dementia (OR = 2.3; 95% CI 1.4-4.0), and hypertension was a risk factor for psychiatric admission (OR = 2.2; 95% CI 1.2-4.1) while peripheral vascular disease was inversely associated (OR = 0.36; 95% CI .14-.87). Age and race/ethnicity were not significantly related. Psychiatric diagnoses were related as expected, especially alcohol dependence (OR = 5.2, 95% CI 2.6-10.0) and schizophrenia (OR = 3.5; 95% CI 1.7-7.3). Details are reported in Table 4.

**Table 4:** Factors Associated with Psychiatric Admissions among Geriatric Patients with New-Onset Epilepsy (N = 1,610; c-statistic = 0.83).

Effect	Odds Ratio	95% CI	Significant (CI excludes 1.0)
Alcohol dependence	5.17	2.66-10.1	*
Schizophrenia	3.38	1.63-7.01	*
Bipolar disorder	2.79	1.12-6.96	*
Dementia	2.24	1.32-3.81	*
Hypertension	2.14	1.16-4.05	*
Depression	2.14	1.27-3.62	*
PTSD	2.10	0.94-4.68	n.s.
Hepatitis	2.03	0.58-7.02	n.s.
Anxiety	1.81	0.96-3.41	n.s.
Other arthritic disease	1.75	0.80-3.83	n.s.
Gout	1.60	0.58-4.41	n.s.
COPD	1.50	0.91-2.49	n.s.
Angina	1.46	0.69-3.08	n.s.
Ulcer peptic	1.44	0.59-3.55	n.s.
Low-back pain	1.43	0.79-2.60	n.s.
Irregular heartbeat	1.39	0.79-2.49	n.s.
Cerebrovascular disease	1.37	0.83-2.26	n.s.
Anemia	1.33	0.76-2.32	n.s.
Diverticulitis	1.32	0.49-3.61	n.s.
Age 75-84 years	1.27	0.77-2.09	n.s.
Neurological conditions, other	1.27	0.63-2.59	n.s.
Hip problems	1.27	0.42-3.79	n.s.
Rheumatoid arthritis	1.27	0.25-6.38	n.s.
Heart attack	1.26	0.61-2.87	n.s.
Lost to follow-up	1.24	0.65-2.37	n.s.
African American	1.22	0.67-2.20	n.s.
Enlarged prostate	1.18	0.70-2.01	n.s.
Cataracts	0.99	0.54-1.79	n.s.
CHF	0.99	0.53-1.86	n.s.
Hispanic	0.98	0.34-2.79	n.s.
Thyroid	0.81	0.34-1.93	n.s.
Gallbladder disease	0.80	0.14-4.55	n.s.
Osteoarthritis	0.77	0.42-1.39	n.s.
Diabetes	0.76	0.44-1.29	n.s.
Cancer	0.64	0.34-1.19	n.s.
Prostatitis	0.60	0.05-7.16	n.s.
Age 85 or older	0.53	0.07-4.19	n.s.
Brain tumor	0.43	0.05-4.09	n.s.
Head injury	0.35	0.04-2.89	n.s.
Peripheral vascular disease	0.34	0.14-0.84	*

The model of medical admissions among new-onset epilepsy patients found that both African American race (OR = 2.3; 95% CI 1.7-3.1) and Hispanic ethnicity (OR = 2.4; 95% CI 1.4.4.2) were associated with greater relative odds of admission. The prior neurological factors of cerebrovascular disease (OR = 1.7; 95% CI 1.3-2.2) and dementia (OR = 1.5; 95% CI 1.1-2.1) were significant. Among physical disorders, most strongly related were myocardial infarction (OR = 4.7; 95% CI 2.7-8.3) and arrhythmias (OR = 2.3; 95% CI 1.7-3.1). Finally, the mental disorder alcohol dependence was again correlated with medical admission (OR = 2.5; 95% CI 1.5-4.1; Table 5).

**Table 5:** Factors Associated with Medical Admissions among Geriatric Patients with New-Onset Epilepsy (N = 1,610; c-statistics = 0.80).

Effect	Odds Ratio	95% CI	Significant (CI excludes 1.0)
Heart attack	4.74	2.72-8.28	*
Gallbladder disease	3.90	1.21-12.58	*
Anemia	2.93	2.09-4.10	*
Angina	2.57	1.57-4.22	*
Alcohol dependence	2.46	1.49-4.08	*
Head injury	2.44	0.88-6.81	n.s.
Hispanic	2.35	1.35-4.09	*
Brain tumor	2.30	0.74-7.14	n.s.
Irregular heartbeat	2.26	1.66-3.08	*
African American	2.25	1.67-3.05	*
Lost to follow-up	2.12	1.53-2.94	*
Cancer	2.08	1.56-2.78	*
Hip problems	2.05	0.98-4.27	n.s.
Thyroid	1.87	1.15-3.03	*
Ulcer peptic	1.73	0.94-3.16	n.s.
Cerebrovascular disease	1.72	1.34-2.19	*
COPD	1.66	1.27-2.18	*
Schizophrenia	1.65	0.93-2.90	n.s.
Peripheral vascular disease	1.61	1.11-2.34	*
Dementia	1.54	1.12-2.12	*
Enlarged prostate	1.53	1.16-2.02	*
Hypertension	1.51	1.15-1.98	*
Diabetes	1.46	1.13-1.89	*
Rheumatoid arthritis	1.46	0.59-3.64	n.s.
CHF	1.44	1.02-2.05	*
Neurological conditions, other	1.39	0.92-2.10	n.s.
Anxiety	1.29	0.84-1.98	n.s.
Prostatitis	1.27	0.41-3.93	n.s.
Other arthritic disease	1.22	0.75-1.99	n.s.
Osteoarthritis	1.16	0.86-1.56	n.s.
Gout	1.11	0.59-2.07	n.s.
Bipolar disorder	1.11	0.55-2.25	n.s.
Cataracts	1.09	0.81-1.47	n.s.
PTSD	1.07	0.60-1.91	n.s.
Depression	1.00	0.72-1.39	n.s.
Hepatitis	0.98	0.37-2.62	n.s.
Age 85 or older	0.90	0.46-1.78	n.s.
Low-back pain	0.89	0.63-1.25	n.s.
Diverticulitis	0.84	0.46-1.53	n.s.
Age 75-84 years	0.81	0.64-1.04	n.s.

## Discussion

**Figure 1:**
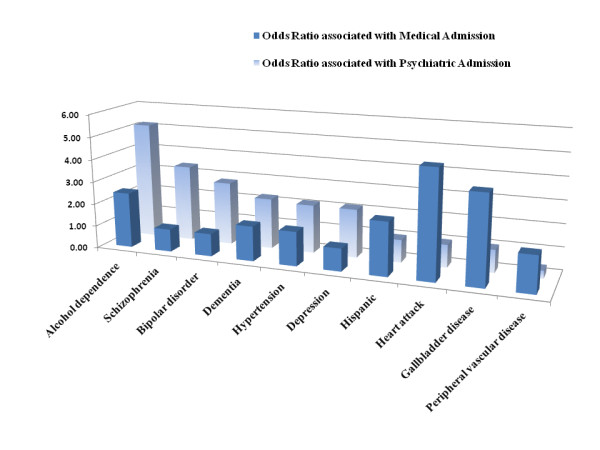
Selected Factors Contrasting Odds Ratios Associated with Medical Admission and Those Associated with Psychiatric Admission among Geriatric Patients with New-Onset Epilepsy (OR = 1 is n.s.)

To our knowledge, this is the first paper to examine the high rates of hospital admissions among elderly new-onset epilepsy patients (52%), relative to elderly patients without epilepsy (15%). Results suggest that the diagnosis of epilepsy in this population signifies a risk not only for recurrent seizures but also for hospitalization for a diversity of medical and psychiatric conditions. In this large sample of older patients using the VA healthcare system, those with new-onset epilepsy had more admissions compared to similar patients without epilepsy. Our study also highlights the association of multiple comorbidities and race/ethnicity with hospitalization among older veterans, and, strikingly, of alcohol dependence (see Figure 1). Alcohol dependence in the elderly is under-diagnosed yet a major factor in hospitalizations in this age group [18].

Echoing prior population-based studies, TIGER patients with new-onset epilepsy showed more than doubled rates of cerebrovascular disease, dementia, and psychiatric disorders - conditions associated with or sharing etiology with epilepsy. Future studies could examine which medical conditions present at the onset of epilepsy may contribute more to the increase in admissions, and how these affect a patient's course of treatment. In this elderly sample, epilepsy imparted an increased risk of both medical and psychiatric admission by a factor of 3 and 6, respectively.

The incidence of epilepsy noted among these VA patients aged 66 years or older (156 cases/100,000 patients) is well above the range in population-based studies reviewed by Banerjee and colleagues, 16-51 per 100,000 [3]. The high incidence in this VA sample may be attributable to their elderly age, patient status, and to the generally high comorbidity seen in the VA patient population [19]. The VA preferentially provides care to veterans who are sicker, more disabled, impoverished, or combat-exposed.

The findings regarding race/ethnicity are of note. Unexpectedly, minority race/ethnicity patients with new-onset epilepsy were at higher risk for hospitalization for medical reasons compared to patients not identified as white. At the same time, Hispanic ethnicity was associated with less risk of psychiatric admission in the total-sample analysis (but not in the new-onset epilepsy sub-analysis). In other words, non-white patients had relatively higher risk of medical admission, and the effect was stronger for non-white patients with new-onset epilepsy. The Selim physical comorbidity scores for Hispanic patients (2.8 +/- 1.9) were marginally lower than those of white patients (2.9 +/- 2.0); African-American patients had slightly higher scores (3.3 +/- 2.1) and were also more likely to have new-onset epilepsy. Whether non-white patients have less physiological reserve to cope with new-onset epilepsy, or differences in the specific comorbid conditions account for admission differences, is unclear.

TIGER patients hospitalized with a primary diagnosis of psychiatric disorder, epilepsy, or non-epilepsy medical conditions had notable differences in neurological and psychiatric comorbidity. A new diagnosis of epilepsy in a geriatric patient should not be viewed as an event easily dismissed with the prescription of an anti-epileptic drug. Rather the clinician should view this development as one attended by high risk of hospitalization and associated with a complex care plan. Furthermore, this may be a signal to consider caretaker availability, health education needs, and potentially end-of-life planning. In addition, limitations in patient-provider communication often arise for mental health patients, such that recognition of medical needs may be impeded in psychiatric patients. Clinicians who are not mental health specialists may want to consult with a geriatric psychiatrist to optimize a coordinated treatment plan, while mental health professionals will want to establish cooperative relationships with the medical specialists.

Comorbidity in epilepsy patients has been studied in only a few large studies, notably the Canadian and United Kingdom studies already cited, where high rates of digestive system disorders, stroke, respiratory disorders, dementia, migraine and other pain conditions as well as doubled co-occurring psychiatric disorders were noted [2,4]. VA patients are generally sicker than US residents [19]. The "protective" effect of peripheral vascular disease, which was inversely associated with psychiatric hospitalization among new-onset epilepsy, and the few other inversely related associations noted in the geriatric population (tables 2 and 3), merit further research but may be artifacts of the analysis, which estimated effects for a very large number of conditions simultaneously.

Limitations of this study include reliance on administrative data, gathered for purposes other than the current study and lacking in many measures that might be of interest, such as severity of illness scales and subjective experience reports. An additional limitation is uncertainty regarding the onset of epilepsy, as a person may have a seizure disorder for some time before it is recognized by diagnosis and treatment. Finally, the geriatric VA patient population is primarily male, reflecting military recruitment patterns, and VA patients are less healthy and wealthy than US residents in general [19], thus these results may not generalize to healthier patients nor to women.

## Conclusion

Our findings highlight the role psychiatric and physical conditions play in both medical and psychiatric hospitalizations. Regardless of the type of hospitalization, substance abuse, dementia and cerebrovascular disease were strong risk factors for admission. Recognition of medical and mental health comorbidities is essential for appropriate treatment on both an inpatient and outpatient basis, and research is needed to determine whether race/ethnicity may play a role in the likelihood of admission type, considering that there may be underlying medical or equity issues to disentangle.

In assessing the inpatient care required by elderly VA patients, our examination of medical and psychiatric admissions found both considerable overlap between groups characterized by psychiatric and neurological conditions, and a large impact on inpatient utilization of epilepsy. From the patient point of view, having epilepsy appeared to increase the risk of medical admission as well as of psychiatric admission. From an analytic perspective, omitting epilepsy and other neurological conditions may lead to overestimation of the risk of admission attributable solely to psychiatric or common medical conditions. Finally, evaluated from a health systems, policy and resource allocation perspective, the emerging picture of the psychiatric epilepsy patient, characterized by a great need for healthcare resources, may merit development of practice guidelines to improve coordinated outpatient care and reduce demand on the healthcare system. As the Baby Boomer cohort ages into late life over the next two decades, this patient population and its healthcare needs are likely to expand greatly.

## Competing interests

The authors declare that they have no competing interests.

## Authors' contributions

LAC conceptualized the study, conducted the analyses, drafted and finalized the manuscript. JEZ contributed to the conceptual framework and writing. ABE provided clinical insight on issues regarding epilepsy and wrote portions of the final manuscript. JMG provided clinical insight on issues regarding psychiatric disorders and wrote portions of the final manuscript. MJP conceived, obtained funding for, and conducted the overall study, and contributed to interpreting study results, writing and finalizing the manuscript. All authors read and approved the final manuscript.

## Pre-publication history

The pre-publication history for this paper can be accessed here:

http://www.biomedcentral.com/1472-6963/11/84/prepub
